# A narrative review of diaphragm ultrasound to predict weaning from mechanical ventilation: where are we and where are we heading?

**DOI:** 10.1186/s13089-019-0117-8

**Published:** 2019-02-28

**Authors:** Peter Turton, Sondus ALAidarous, Ingeborg Welters

**Affiliations:** 1 Critical Care Unit, Royal Liverpool University Hospital, Liverpool, UK; 2 Institute of Aging and Chronic Disease, University of Liverpool, Liverpool, UK; 3 Institute of Infection and Global Health, University of Liverpool, Liverpool, UK

**Keywords:** Diaphragm, Muscle atrophy, Diaphragm ultrasound, Thickening fraction

## Abstract

**Background:**

The use of ultrasound to visualize the diaphragm is well established. Over the last 15 years, certain indices of diaphragm function, namely diaphragm thickness, thickening fraction and excursion have been established for mechanically ventilated patients to track changes in diaphragm size and function over time, to assess and diagnose diaphragmatic dysfunction, and to evaluate if these indices can predict successful liberation from mechanical ventilation. In the last 2 years, three meta-analyses and a systematic review have assessed the usability of diaphragmatic ultrasound to predict successful weaning. Since then, further data have been published on the topic.

**Conclusions:**

The aim of this narrative review is to briefly describe the common methods of diaphragmatic function assessment using ultrasound techniques, before summarizing the major points raised by the recent reviews. A narrative summary of the most recent data will be presented, before concluding with a brief discussion of future research directions in this field.

## Background

There has been much interest in the use of diaphragm ultrasound as a tool of measuring and tracking atrophy, in particular to identify patients who will wean from mechanical ventilation, and who will remain free of ventilatory support afterwards. Two meta-analyses and a systematic review have been published on the topic in the last 2 years, and more work is being produced. The aim of this narrative review is briefly re-iterate what is being measured with diaphragm ultrasound, to summarize the most recent findings from these reviews and meta-analyses, and to provide an update of current work produced after these reviews.

## The diaphragm in critical care: what do we know?

The effects of atrophy of the diaphragm secondary to mechanical ventilation have been recently described; Goligher found that the development of diaphragm atrophy was associated with prolonged duration of mechanical ventilation, increased ICU length of stay, and a higher rate of complications [1]. Interestingly, patients who showed an increase in diaphragmatic thickness during their critical illness were also at higher risk of prolonged mechanical ventilation, with excessive respiratory effort as a possible underlying trigger. The authors did acknowledge that tissue oedema from fluid resuscitation may also contribute to this thickening. Diaphragmatic thickness has been shown to reduce by 6% [2] or 7.5% [3] per day in mechanically ventilated patients. However, a further study demonstrated that although nearly half of the patients in their study did suffer atrophy, the same proportion experienced no loss, and a further 10% actually had increases in diaphragmatic thickness [4]. A recent study in mechanically ventilated children suggested that diaphragmatic atrophy occurs at an average rate of 3.4% per day and is worsened by the use of neuromuscular blockade [5]. However, two papers failed to demonstrate diaphragmatic atrophy using ultrasound [6, 7]. However, one of these studies was in extubated survivors of sepsis (82% of which had either severe sepsis or septic shock) who were approached after a period of at least 5 days of mechanical ventilation, compared to controls. However, the authors concede that these results were based on a single measurement at a point in the patients’ recovery from sepsis rather than during the acute episode.

## Ultrasound and the diaphragm

**Fig. 1:**
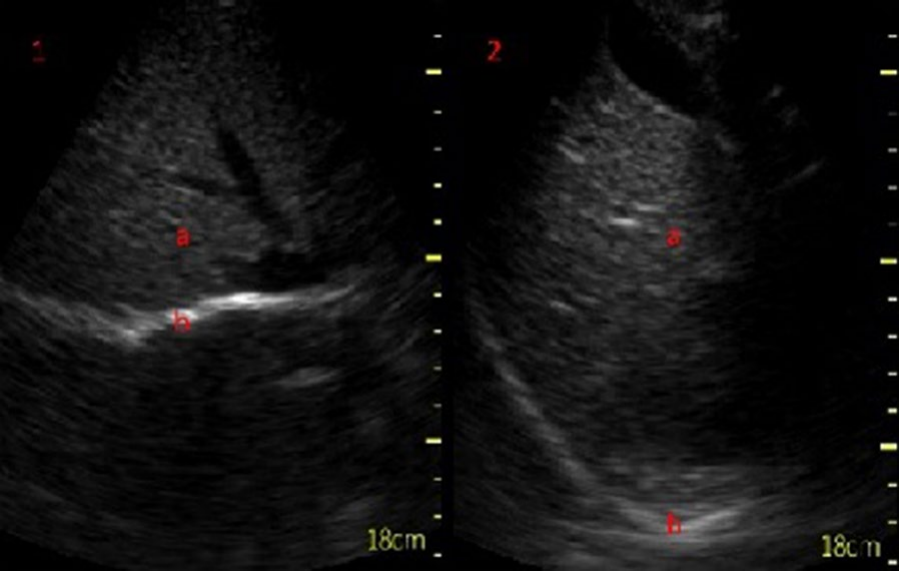
Subcostal view of the diaphragm (b) in B-mode at end inspiration (1) and at expiration (2) seen below the liver (a)

**Fig. 2:**
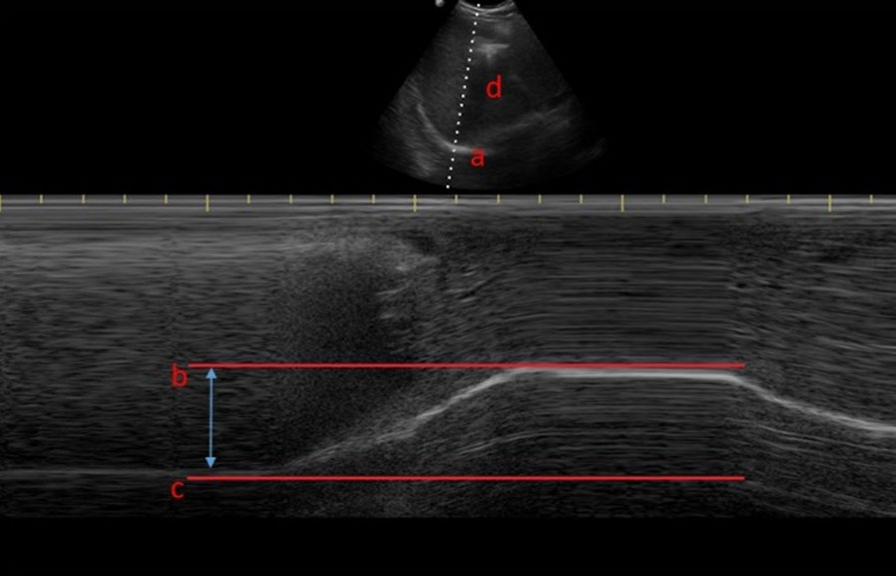
Diaphragm excursion as assessed via M-mode ultrasonography, where a is the diaphragm, b is at the end of a deep inspiratory effort, c is at end expiration and d is the liver

**Fig. 3:**
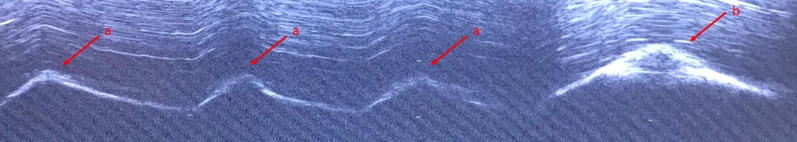
M-mode ultrasonography demonstrating three tidal inspiratory efforts (**a**) and a deep inspiratory effort (**b**)

Visualization of the diaphragm with ultrasound has been possible for well over 40 years [8]. However, only recently diaphragmatic ultrasound has been used to assess diaphragm function and size during mechanical ventilation. There are two commonly used measurements derived from ultrasound: diaphragm excursion and diaphragm thickness [9]. Diaphragm excursion is usually measured using a phased array probe, with the probe positioned in the subcostal margin in the mid-clavicular line, with the aim of imaging the posterior third of the diaphragm (Fig. 1). Although some studies have used B-mode imaging to determine diaphragmatic excursion [10], the use of M-mode produces images that visualize the movement of the diaphragm over time and allows accurate measurement of diaphragmatic displacement over a respiratory cycle (Figs. 2 and 3) [11]. In healthy volunteers, diaphragmatic excursion is known to vary with sex and height and can be reliably performed in a recumbent or supine position [12]. Excursion is known to positively correlate with lung inspiratory volumes [13, 14], and is higher during forced inspiratory breathing [10].

**Fig. 4:**
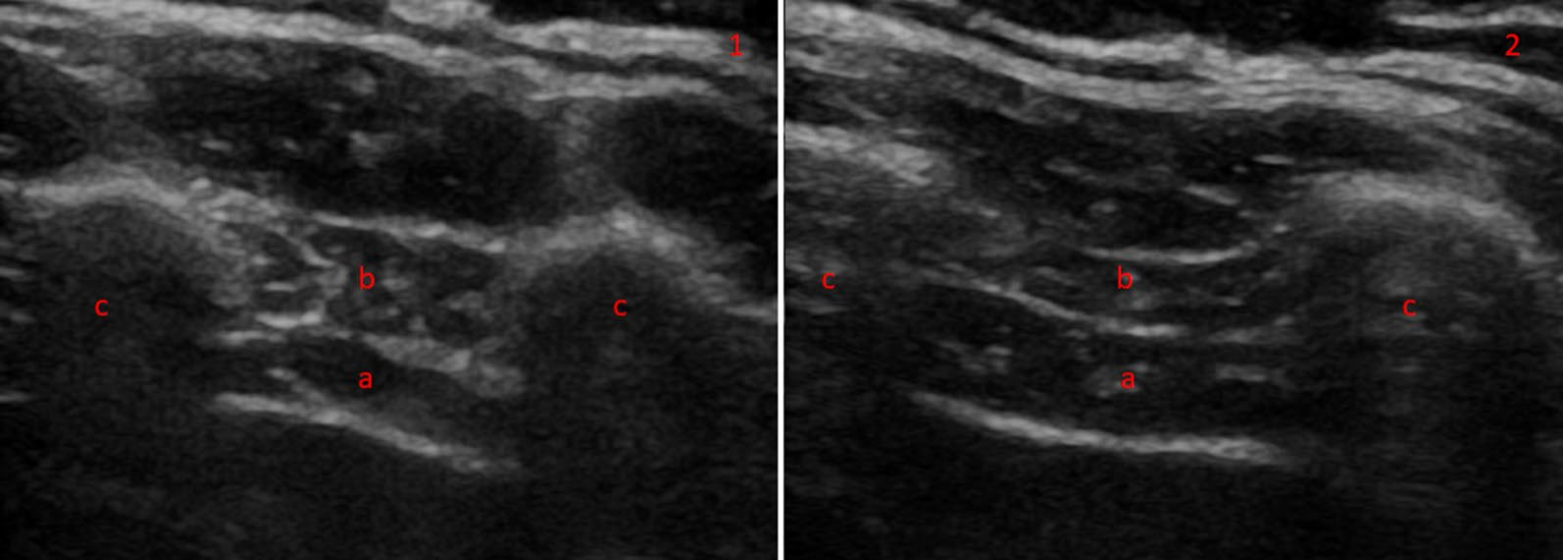
Diaphragm thickness in B-mode thoracic view at end expiration (1) and inspiration (2) in a heathy volunteer. The diaphragm can be seen between two echogenic layers (a) with the intercostal compartment above (b). The two muscle layers sit between two ribs (**c**)

Diaphragm thickness is measured in the zone of apposition, using a higher-frequency (> 10 MHz) linear probe, to view the diaphragm as a three-layered structure, sandwiched between the two echogenic layers of the pleura and the peritoneum (Fig. 4) [15]. Both B- and M-mode techniques can be used to measure thickness [16]. Diaphragm thickness has been previously correlated with the strength of the diaphragm [17], but not the endurance or fatigability [18]. It appears to be thicker in an upright position, compared to supine posture [19], can be measured at expiration or end inspiration, and in both tidal and maximal breathing. Comparing expiratory with inspiratory thickness gives the thickening fraction, which is usually denoted as [(End Inspiratory Thickness − End Expiratory Thickness)/End Expiratory Thickness] [20] and is an indicator of the work of breathing [21]. These measurements can be used to form a definition of diaphragm dysfunction, although there is variation in this definition: It has been defined as a thickening fraction of less than 20% or a tidal excursion of less than 10 mm [22], based on the presence of paradoxical movement in the case of the paralyzed diaphragm [9], or using non-ultrasound methods such as measurement of twitch pressures [23]. Regardless, ultrasound techniques have been shown to outperform traditional techniques such as fluoroscopy in diagnosing diaphragm dysfunction [24].

## Summary of current literature

In 2017, a systematic review [25] and a meta-analysis [26] have been performed, assessing the evidence on diaphragm ultrasound and its ability to predict successful weaning from mechanical ventilation. Two further meta-analyses have been published in 2018 [27, 28], and together these reviews assessed the combined work of more than 30 individual papers (excluding further 3 papers that looked exclusively at lung rather than diaphragm ultrasound).

The systematic review [25] focused on the use of diaphragmatic ultrasound in four key areas: to diagnose diaphragmatic dysfunction, to predict successful weaning from mechanical ventilation, to determine if ultrasound can assess muscular workload against other known measurements such as transdiaphragmatic pressure [29], and to describe variations in diaphragm atrophy across studies.

With respect to weaning from mechanical ventilation, four studies were analyzed, two of which described diaphragm excursion either by M-mode ultrasound [30] or by measuring organ displacement [31]. The two remaining studies assessed diaphragmatic thickening fraction [32, 33]. All four studies concluded that their respective measurements can predict successful extubation or weaning failure, with cut-off values of 11–14 mm in excursion and 30–36% in thickening fraction being most sensitive and specific.

The three meta-analyses are broadly similar in their aims and results. A possible reason may lie in the slight differences to the selection criteria; Li et al. reviewed only publications in English and defined weaning failure as the requirement for re-intubation within 48 h, whereas Llamas-Álverez et al. included publications in all languages and had a much broader definition of weaning failure to include death, unscheduled non-invasive ventilation, tracheostomy formation or the failure of a spontaneous breathing trial within 72 h, and Qian defined weaning failure more broadly as a failed spontaneous breathing trial, re-intubation, the use of non-invasive ventilation, or death. Li and Llamas-Álverez found similar AUC characteristics for the use of diaphragm thickening fraction (0.83 versus 0.87). Llamas-Álverez et al. concluded that thickening fraction may help to predict weaning failure, and Li et al. concluded that the either measurement is suitable to predict successful extubation. Qian found that pooled specificity for predicting weaning success was similar to the work of Llamas-Álverez, and also found that weaning failure was higher in the presence of diaphragmatic dysfunction, and that both excursion and thickening fraction were higher in patients who were successfully weaned.

All papers acknowledge the heterogeneity in the studies analyzed. The definition of weaning failure varied amongst individual studies, with re-intubation limits set at either 48 or 72 h, and some studies included the use of non-invasive ventilation to define weaning failure. Inclusion criteria are different for each of the studies; while some studies recruited patients during their first spontaneous breathing trial [32], another study included only patients who had already had a failed trial [33]. Further differences arise from the ultrasonic technique chosen; in Li’s meta-analysis, 4 of the studies were conducted with the patient in a supine position, while patients were semi-recumbent in the remaining 9 studies. Although probe position was consistent in all studies, probe frequency and ultrasound machine manufacturer varied considerably, with 12 different types of ultrasound machines being used, covering a range of frequencies from 3.5 to 10 MHz. There is also variation in the patient populations, particularly with respect to age and sex. It is known that age negatively correlates with excursion in deep breathing, and that females have less diaphragmatic excursion [12]. Many of the studies included both left and right sides of the diaphragm, but some reported measurements of the right side only, probably because there is greater difficulty in imaging the left diaphragm due to the lung obscuring the view [13]. Finally, there is variation regarding the time point during a spontaneous breathing trial at which measurements are taken, with ultrasound images being obtained at the start or end of spontaneous breathing; some investigators assessed diaphragmatic function after extubation, others during mechanically ventilation with further variation in the ventilatory mode used. It has been suggested that pre-extubation is the best time to perform diaphragm ultrasound to assess the diaphragm at a time point when it may be fatigued. A protocol for a new study performing ultrasounds at regular intervals throughout 2 h of spontaneous breathing has been published, but the results are not yet available [34].

## Where are we now?

Since the publication of the systematic reviews, several studies have reported diaphragmatic ultrasound parameters as a means of predicting extubation success. Our search strategy used MEDLINE only; we searched for papers published after 1st January 2017 until to the current date of writing (August 2018). Using the search string (Ultraso* AND Diaphagm* AND (critical* OR intensive OR sepsis OR mechanical * OR ventilat*) yielded 89 results. After having excluded papers that already appeared in the four systematic reviews and papers covering different topics as assessed on title and/or abstract), we identified 18 new papers since 2017 that have not been part of a systematic review.

## Newer studies

Newly identified studies can be divided into three categories. In the first, there are studies in which diaphragm thickening or excursion are used alone to predict successful weaning. In the second, diaphragm ultrasound is compared to another technique; and in the third, these techniques are combined to see if they increase predictive accuracy.

A recent study describes cut-offs for diaphragm thickening to predict successful weaning *prior* to a spontaneous breathing trial [35]. Its results are in keeping with previously established cut-offs for patients weaning from pressure support [36]. However, there are also conflicting results. For example, a study evaluating diaphragm excursion using spleen and liver displacement found that displacement of the organs by 1.2 cm was the best cut-off for predicting successful extubation [37]. However, poor agreement between solid organ movement and diaphragm excursion has been described before [38]. Another recent study found that diaphragmatic excursion, and not thickening fraction, was the best predictor of extubation failure in patients undergoing their first spontaneous breathing trial [39]. The most recent reliability study has established values of inter- (0.987) and intra-observer variability (0.986) that are within the higher range of Intra Class Correlation (ICC) coefficients established in the systematic review [40].

## Newer studies—comparative approaches

Dres and colleagues compared the performance of diaphragm ultrasound against tracheal pressure measurements obtained during supra-maximal phrenic nerve stimulation during a spontaneous breathing trial [23]. They not only found that a lower stimulated pressure than previously accepted was associated with optimum sensitivity and specificity for liberation from mechanical ventilation [41], but also described that a thickening fraction of greater than 25.8% gave equivalent accuracy of prediction in comparison to phrenic nerve stimulation, with AUC–ROC values of 0.80 and 0.82 for phrenic nerve stimulation and diaphragm thickening fraction, respectively.

## Newer studies—combined approaches

Combining diaphragmatic ultrasound with echocardiography may be a promising route for prediction of successful weaning, particularly in view of potential cardiac causes for a failed respiratory wean [42]. The ratio of mitral Doppler inflow velocity (E) to annular tissue Doppler wave velocity (Ea, *E*/Ea ratio) has been measured with transthoracic echocardiography alongside diaphragmatic excursion in patients who were extubated after a successful spontaneous breathing trial (SBT) [43]. The authors found that respiratory failure within 48 h of extubation could be predicted from both *E*/Ea and left ventricular ejection fraction values, but that reintubation within a week of extubation was more accurately predicted by diaphragmatic excursion.

Another study combined echocardiography with lung ultrasound and assessment of diaphragmatic excursion to assess if all three combined could predict extubation failure in patients undergoing a trial of pressure support ventilation [44]. The results were confirmed in a smaller sub-study of patients breathing via a T-tube, although out of the three modalities, diaphragm ultrasound contributed least predicting successful weaning. Furthermore, a recent small observational study has combined echocardiography and lung ultrasound for assessment of aeration with diaphragmatic ultrasound, and reported that lung aeration and markers of diastolic dysfunction were the only strong predictors of successful extubation [45].

Another combined approach combined diaphragm thickening fraction with the Rapid Shallow Breathing Index (RSBI). First described in 1991 [46], RSBI is defined as the ratio of the respiratory frequency to the tidal volume [47], with a cut-off value of 100–105 breaths/min/liter being associated with successful extubation [46, 48]. A recent study found that RSBI alone, in comparison to measurements derived from diaphragm ultrasound, was most accurate in predicting success of extubation, with an ROC–AUC of 0.96 and a sensitivity and specificity of 100% [49]. This supports earlier work that the sensitivity, specificity and positive predictive value of a thickening fraction cut-off 36% were comparable to RSBI, but ultimately lower than it [33]. However, the combination of RSBI with diaphragm thickening fraction of greater than 26% was a more accurate predictor of successful weaning from mechanical ventilation than RSBI alone [50]. The authors concluded that thickening fraction of the right diaphragm alone was as accurate as this combined approach, and suggested that thickening fraction could replace RSBI as the most commonly used weaning parameter in the future.

## Future directions

As ultrasound technology progresses, it may be possible for clinicians to estimate diaphragm thickness and excursion using portable, hand-held devices. A recent study used both linear and phased arrays probes of a hand-held ultrasound device to assess diaphragmatic thickness and excursion, respectively, compared to a standard ultrasound device [22]. Good agreement was noted between the two devices, with ICCs of greater than 0.9 noted in all indices of measurement except for maximal excursion. Based on a definition of diaphragmatic dysfunction as tidal excursion of less than 10 mm, the detection of dysfunction was comparable between the two devices, and good inter-rater reliability was also seen. Stronger agreement between the two devices was seen in the measurement of diaphragm thickness compared to measurement of excursion, possibly because of the hand-held device lacking an M-mode for accurate measurement of excursion.

A third measurement, the contraction velocity, has also been evaluated recently. Contraction velocity is an extension of diaphragm excursion, dividing excursion by the time to reach maximal excursion [9]. None of the systematic reviews assessed contraction velocity in the prediction of successful weaning. A study of elderly ventilated patients found that right-sided contraction velocity had a similar AUC as right-sided excursion (labeled in the study as diaphragmatic motion), and that both of these were more predictive for successful weaning from mechanical ventilation than RSBI [51]. Contraction velocity has been shown to have high sensitivity and specificity, and only performed slightly worse than RSBI in a study of younger patients [49]. A more recent study, however, found that there was no difference in contraction velocity between patients who were successfully extubated, compared to those who were re-intubated [52]. It is not clear why there is such variation in results, and further research on conduction velocity is required, along with standardization whether velocity is measured over tidal or maximal inspiratory efforts. The same authors found that multiplying the diaphragmatic excursion (*E*) by the inspiratory time (*I*) to give a diaphragmatic excursion-time index gave values that were significantly higher in patients who had been successfully extubated compared to those who failed extubation [53]. The differences were still significant regardless as to whether the measurements were performed during spontaneous breathing or after extubation. However, significance was lost during pressure-assist ventilation modes.

Speckle tracking can detect tissue motion and distortion [54]. In healthy volunteers, it has been used to successfully assess diaphragmatic strain under pressure support ventilation [55] and was weakly but significantly associated with caudal diaphragm displacement [56]. This technique may provide useful information about the diaphragm during controlled mechanical ventilation, but as yet, there are no studies examining speckle tracking in the critical care population. Similarly, an “area method” [57] assessing diaphragm motion in two dimensions, correlates with lung volume using both B and M-mode ultrasound in healthy volunteers, and can be performed on both sides of the chest.

Further research focuses on the prediction of successful extubation in particular patient groups. For example, a recent study demonstrated that diaphragm thickness measured before anesthetic induction correlates with time to extubation in patients undergoing liver transplants. Time to extubation after the procedure was higher in patients with pre-operative end expiratory diaphragm thickness of less than 2 mm [58].

In patients with Chronic Obstructive Pulmonary Disease (COPD), diaphragmatic ultrasound may predict successful weaning on one side, but could also serve to predict success of non-invasive ventilation. In this context, it has been reported that COPD patients with diaphragm dysfunction (diagnosed by a thickening fraction of less than 20%), who require non-invasive ventilation, were 4.4 times more likely to need intubation, more often proceeded to tracheostomies, and had increased length of stay in ICU and hospital mortality [59]. These results were in line with an earlier smaller study that also described an association of diaphragm dysfunction with Non-Invasive Ventilation (NIV) failure and increased mortality [60]. However, reduced diaphragmatic thickening itself is not a risk factor for acute exacerbation of COPD [61].

## Conclusions

Diaphragmatic ultrasound has been extensively studied as a predictor of successful weaning from mechanical ventilation, and continues to be studied. It remains difficult to draw general conclusions from individual studies due to the marked variation in study design and population. Even, definitions such as a failed breathing trial or failed extubation have not been standardized across studies, rendering comparison between outcome measures impossible. As yet, defined cut-offs for measurements of diaphragmatic ultrasound have been agreed, and there are no randomized control trials available. Although diaphragmatic ultrasound is a promising diagnostic tool, greater standardization of protocols, outcome measures and ventilatory settings is required for further research and clinical application.

## Abbreviations

AUC: area under the curve
AUC–ROC: area under the curve (receiver-operator characteristic)
COPD: Chronic Obstructive Pulmonary Disease
ICC: intra-class correlation coefficient
ICU: intensive care unit
NIV: non-invasive ventilation
SBT: spontaneous breathing trial
RSBI: Rapid Shallow Breathing Index
